# DNA barcodes and phylogenetic affinities of the terrestrial slugs *Arion gilvus* and *A. ponsi* (Gastropoda, Pulmonata, Arionidae)

**DOI:** 10.3897/zookeys.365.6104

**Published:** 2013-12-30

**Authors:** Karin Breugelmans, Kurt Jordaens, Els Adriaens, Jean Paul Remon, Josep Quintana Cardona, Thierry Backeljau

**Affiliations:** 1 Royal Belgian Institute of Natural Sciences, OD Taxonomy and Phylogeny (JEMU), Vautierstraat 29, B-1000 Brussels, Belgium; 2 Royal Museum for Central Africa (JEMU), Leuvensesteenweg 13, B-3080 Tervuren, Belgium; 3 Evolutionary Ecology Group, University of Antwerp, Groenenborgerlaan 171, B-2020 Antwerp, Belgium; 4 Laboratory of Pharmaceutical Technology, University of Ghent, Harelbekestraat 72, B-9000 Ghent, Belgium; 5 Institut Catala de Paleontologia Miquel Crusafont, Universitat Autònomo de Barcelona, edifici ICP Campus de la UAB, sln 08193 Cerdanyola del Vallès, Barcelona, Spain

**Keywords:** DNA barcoding, terrestrial slugs, Gastropoda, taxonomy, Iberian Peninsula, *Arion ponsi*, *Arion gilvus*

## Abstract

The Iberian Peninsula is a region with a high endemicity of species of the terrestrial slug subgenus *Mesarion*. Many of these species have been described mainly on subtle differences in their proximal genitalia. It therefore remains to be investigated 1) whether these locally diverged taxa also represent different species under a phylogenetic species concept as has been shown for other *Mesarion* species outside the Iberian Peninsula, and 2) how these taxa are phylogenetically related. Here, we analysed DNA sequence data of two mitochondrial (COI and 16S) genes, and of the nuclear ITS1 region, to explore the phylogenetic affinities of two of these endemic taxa, viz. *Arion gilvus* Torres Mínguez, 1925 and *A. ponsi* Quintana Cardona, 2007. We also evaluated the use of these DNA sequence data as DNA barcodes for both species. Our results showed that ITS did not allow to differentiate among most of the *Mesarion* molecular operational taxonomic units (MOTUs) / morphospecies in *Mesarion*. Yet, the overall mean p-distance among the *Mesarion* MOTUs / morphospecies for both mtDNA fragments (16.7% for COI, 13% for 16S) was comparable to that between *A. ponsi* and its closest relative *A. molinae* (COI: 14.2%; 16S: 16.2%) and to that between *A. gilvus* and its closest relative *A. urbiae* (COI: 14.4%; 16S: 13.4%). Hence, with respect to mtDNA divergence, both *A. ponsi* and *A. gilvus*, behave as other *Mesarion* species or putative species-level MOTUs and thus are confirmed as distinct ‘species’.

## Introduction

The genus *Arion* Férussac, 1819 is the most species rich genus of the terrestrial slug family Arionidae (Mollusca, Pulmonata, Gastropoda). It comprizes approximately 40 species, grouped into four subgenera, viz. *Arion* s.s. Férussac, 1819, *Kobeltia* Seibert, 1873, *Carinarion* Hesse, 1926 and *Mesarion* Hesse, 1926. Species of the subgenus *Mesarion* (type species: *Limax subfuscus* Draparnaud, 1805) are characterized by 1) a medium body-size (up to 75 mm when extended), 2) an orange to dark brown dorsum, 3) two dark bands on the sides of the mantle, 4) (usually) yellow to orange body mucus, and 5) an enlarged free-oviduct with a long and V-shaped ligula (Kerney et al. 1983). Many *Mesarion* species are highly polymorphic with respect to body colour and genital anatomy. As a consequence, the species limits and phylogenetic relationships of taxa within this subgenus have been debated for decades (e.g. Garrido et al. 1995, Castillejo 1997, 1998, Pinceel et al. 2004, 2005a, b, Quinteiro et al. 2005). *Arion subfuscus* (Draparnaud, 1805) (type locality: Montagne Noire, France) is probably the most problematic “species” within *Mesarion* as it shows an overwhelming amount of variation in body pigmentation, genital anatomy, and reproductive behavior [see Garrido et al. (1995) and the references listed in their table 1]. This variation often has been interpreted as indicating reproductive isolation between geographically isolated populations, and *Arion subfuscus* thus is considered a species complex (Wiktor 1973, Waldén 1976, De Winter 1986, Backeljau 1989, Altonaga et al. 1994, Backeljau et al. 1994, Garrido et al. 1995). Especially in the Pyrenees and the coastal regions of Spain there are local, morphologically diverged populations (e.g. Garrido et al. 1995, Castillejo 1998). Several of these have been described as endemic species on the basis of where the epiphallus, oviduct and pedunculus of the bursa copulatrix open into the atrium, in combination with differences in the relative lengths of the vas deferens and the epiphallus (e.g. Castillejo 1998, Garrido et al. 1995, Quintana Cardona 2007). Two of these endemic taxa occur in the eastern coastal region of Spain or the Balearic Islands, viz. *Arion gilvus* Torres Mínguez, 1925 and *Arion ponsi* Quintana Cardona, 2007.

**Figure 1.**
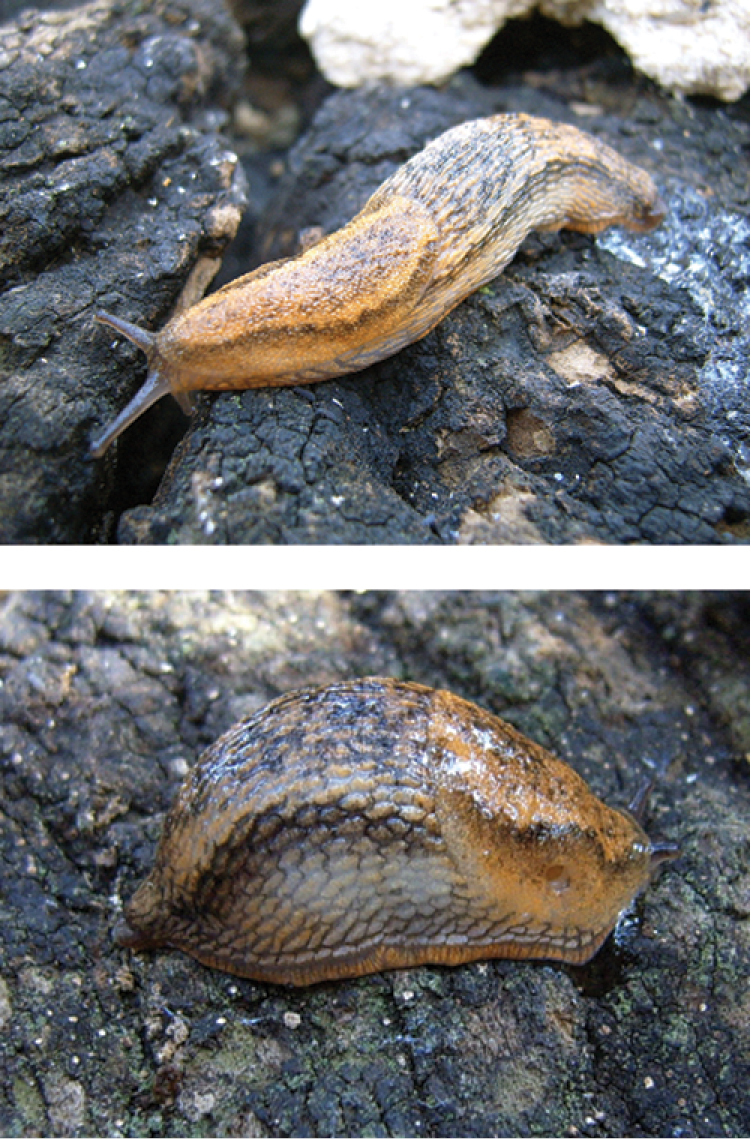
*Arion ponsi* Quintana Cardona, 2007 from Menorca (Balearic Islands, Spain).

*Arion ponsi* (Figure 1) was described from Menorca (Balearic Islands, type locality: Barranc d’Algendar). The species has a medium body size (range: 54–66 mm), an orange to beige dorsal body colour with dark lateral bands that can be blurry in the posterior parts, a foot sole that is cream coloured with a greyish hue, and a transparent body mucus (Quintana Cardona 2007). Its genital anatomy is very similar to that of *Arion gilvus*, *Arion iratii* Garrido, Castillejo & Iglesias, 1995, *Arion molinae* Garrido, Castillejo & Iglesias, 1995 and *Arion lizarrustii* Garrido, Castillejo & Iglesias, 1995, but its epiphallus is shorter than the vas deferens (as in *Arion molinae*) and opens into the genital atrium in between the oviduct and the pedunculus of the bursa copulatrix (unlike in *Arion molinae*, where the pedunculus is positioned in between the epiphallus and oviduct) (figures 3–5 in Quintana Cardona 2007).

**Figure 2.**
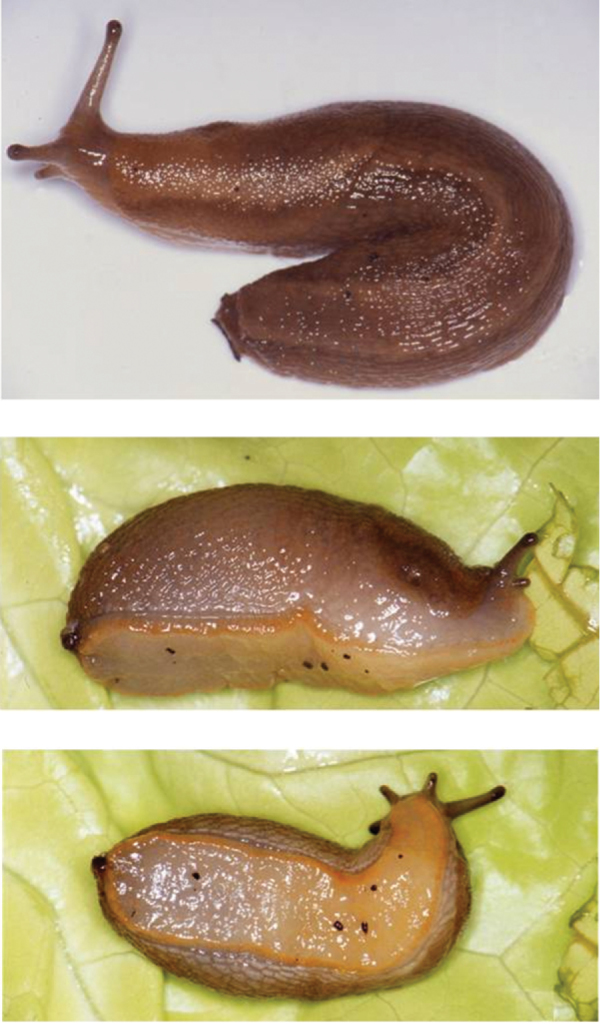
*Arion gilvus* Torres Mínguez, 1925 from Serra de Pandóls (Valencia, Spain). **A** dorsal view **B** lateral view **C** ventral view.

*Arion gilvus* (Figure 2) was described from ‘Mandol’ in the Spanish Province of Tarragona. However, the toponym ‘Mandol’ seems to be erroneous (e.g. Bech 1990) and therefore Castillejo (1990) assigned eight specimens with an *Arion gilvus* morphology from Serra de Pandóls near Gandesa (Province of Tarragona) as topotypes [see also Castillejo and Rodríguez (1991)]. Subsequently, *Arion gilvus* was redescribed by Garrido (1992). Afterwards, the species has also been found in the Provinces of Valencia, Teruel and Albacete [Borredà (1994), figure 15 in Castillejo (1997), figure 1 in Quinteiro et al. (2005)]. *Arion gilvus* reaches a length of up to 65 mm when extended. It has a yellowish to brown dorsum that gets lighter downwards at the sides and dark lateral bands that have a yellowish grey line on their upper side (Figure 1). The sole is white or evenly yellowish and the mucus is pale yellow (Torres Mínguez 1925, Bech 1990, Garrido 1992, Castillejo 1997). The epiphallus, the pedunculus of the bursa copulatrix, and the free oviduct join the atrium on a single line with the pedunculus of the bursa copulatrix in the middle, as in *Arion molinae*, but in contrast to the latter, the epiphallus is longer than the vas deferens (Torres Mínguez 1925, Borredà 1994, Castillejo 1997, and figures 26–28 in Garrido et al. 1995).

As illustrated by *Arion ponsi* and *Arion gilvus*, the alleged species-specific genital differences among the Iberian species of the *Arion subfuscus* complex are very subtle and little is known about their intraspecific variation. Moreover, genital differences among arionid taxa do not necessarily imply reproductive isolation (Dreijers et al. 2013). Hence, if alleged species-specific phenotypic differences in arionids are to be interpreted under a phylogenetic species concept, then their correlation with reproductive isolation should be corroborated by molecular data. Molecular markers have been very effective in this respect (e.g. Pinceel et al. 2005a, b, Quinteiro et al. 2005, Geenen et al. 2006, Jordaens et al. 2010). As such, Quinteiro et al. (2005) investigated the taxonomic affinities of Iberian *Mesarion* species using DNA sequence data. Their analysis of the nuclear ribosomal internal transcribed spacer 1 region (ITS1) showed a polytomy of *Mesarion* species, yet, the analysis of the mitochondrial NADH dehydrogenase I (ND1) gene suggested a strongly bootstrap supported group of Iberian *Mesarion* species with a continental-Mediterranean distribution (*Arion paularensis*, *Arion baeticus*, *Arion urbiae*, *Arion anguloi*, *Arion wiktori*, and *Arion gilvus*), and an unsupported group of species with an Atlantic distribution (*Arion lusitanicus*, *Arion nobrei*, *Arion fuligineus*, *Arion hispanicus* and *Arion flagellus*). In addition, the positions of three Pyrenean species (*Arion lizarrustii*, *Arion iratii*, *Arion molinae*) remained unresolved. More specifically, the ND1 data placed *Arion gilvus* as sister taxon of *Arion urbiae* and *Arion anguloi*. Quinteiro et al. (2005) did not study individuals from the Balearic Islands and thus probably did not include *Arion ponsi*.

Because DNA sequence data do not only provide phylogenetic information, but can also serve as DNA barcodes for species identification (Hebert et al. 2003, 2004), we here expand on the work of Quinteiro et al. (2005) by 1) characterizing *Arion gilvus* and *Arion ponsi* using mitochondrial COI and 16S rDNA gene fragments, and the larger part of the nuclear ITS1 region, 2) exploring the phylogenetic affinities of *Arion gilvus* and *Arion ponsi* within the subgenus *Mesarion*, and 3) providing diagnostic COI barcodes for both species.

## Material and methods

Information on the species and specimens included here is provided in Table 1. In total, we screened 45 specimens (Table 1). DNA was extracted from small parts of the foot using a NucleoSpin Tissue Kit (Macherey-Nagel, Düren) following the manufacturer’s instructions. PCR reactions were done in 25 µl reaction volumes that contained 1.5 mM MgCl_2_ in 1 × PCR buffer (Qiagen), 0.2 mM of each dNTP, 0.2 µM of each primer and 0.5 units of Taq polymerase (Qiagen). A fragment of the mitochondrial COI and 16S genes was amplified using primer pairs LCO1490 and HCO2198 (Folmer et al. 1994) and 16Sar and 16Sbr (Palumbi 1996), respectively. The nuclear ITS1 region (except the ± first 30 bp) was amplified using the primer pair ITS1L and 58C (Hillis and Dixon 1991). The PCR profile was an initial denaturation step of 5 min at 95 °C, followed by 35 cycles of 45 s at 95 °C, 45 s at an annealing temperature of 40 °C (COI), 42 °C (16S) or 55 °C (ITS1) and 1.5 min at 72 °C, and ending with a final extension step of 5 min at 72 °C. PCR products were purified using the GFX PCR DNA Purification Kit (GE Healthcare) following the manufacturer’s instructions. Purified DNA was diluted in 15 µl of sterile water. PCR-products were bidirectionally sequenced using the ABI PRISM BigDye® Terminator v1.1 Cycle Sequencing Kit and run on a ABI3130xl Genetic Analyzer. Sequences were assembled in SeqScape v2.5 (Life Technologies) and inconsistencies were checked by eye on the chromatogram. Sequences were submitted to GenBank under accession numbers KF305196–KF305225 for COI, KF356212–KF356245 for 16S and KF385449–KF385469 for ITS1. These datasets were supplemented with DNA sequences from GenBank [including a few species of the other *Arion* subgenera (Table 1)]. We used those of *Carinarion* as outgroup.

**Table 1.** List of specimens used in this study with specimen ID, sampling locality, GenBank accession numbers for the COI, 16S and ITS1 sequences, and collection number at the museums (if available). Neo-, para- and topotypes have been indicated. Specimen codes with an asterisk are data taken from Quinteiro et al. (2005); NA = not assessed. The specimen ID and GenBank accession numbers of newly sequenced specimens are given in bold.

Species/ID	Locality, country	COI	16S	ITS1	Collection number
**Subgenus *Mesarion* Hesse, 1926**					
*Arion anguloi* Martín and Gómez, 1988					
ang-SU2777	Torralba del Rio, Spain	AY987869	AY947348	AY947386	RBINS Brussels, I.G. 32471
**ang-115** (topotype)	Osma, Álava, Spain	**KF305196**	**KF356212**	AJ509055	RBINS Brussels, I.G. 32471
AANG 73A*	Burgos, Spain	NA	NA	AY316291	
*Arion baeticus* Garrido, Castillejo and Iglesias, 1995					
bae-556 (paratype)	Malaga, Spain	AY987871	AY947350	AJ509054	MNCN Madrid 15.05/6969
*Arion flagellus* Colligne, 1893					
fla-130	Glasgow, UK	AY987880	AY947359	AJ509053	RBINS Brussels, I.G. 32471
fla-161	Glasgow, UK	AY987881	AY947360	AJ509052	RBINS Brussels, I.G. 32471
fla-SU672	Salamir, Spain	AY987882	AY947361	AY947388	RBINS Brussels, I.G. 32471
AFLA 44A*	Croydon, UK	NA	NA	AY316278	
*Arion fuligineus* Morelet, 1845					
AFUL 43A*	São Silvestre, Portugal	NA	NA	AY316277	
*Arion fuscus* (Müller, 1774)					
fus-SU155	Grudki, Poland	AY987885	AJ786721	AY947390	RBINS Brussels, I.G. 32471
fus-2320	Predel, Bulgaria	AY987886	AJ786722	AY947391	RBINS Brussels, I.G. 32471
fus-SU1335	Steinegg, Austria	AY987887	AJ786726	AY947392	RBINS Brussels, I.G. 32471
**fus-SU2188**	Kreuzen, Austria	NA	**KF356221**	NA	RBINS Brussels, I.G. 32471
*Arion gilvus* Torres Mínguez, 1925					
**gil-46**	Serra de Pandóls, Valencia, Spain	NA	NA	**KF385450**	RBINS Brussels, I.G. 32471
**gil-47**	Serra de Pandóls, Valencia, Spain	**KF305199**	**KF356222**	**KF385451**	RBINS Brussels, I.G. 32471
**gil-73**	Serra de Pandóls, Valencia, Spain	**KF305200**	**KF356223**	**KF385452**	RBINS Brussels, I.G. 32471
AGIL 49A*	Serra de Pandóls, Valencia, Spain	NA	NA	AY316282	
*Arion hispanicus* Simroth, 1886					
AHIS 52B*	Cáceres, Spain	NA	NA	AY316285	
*Arion iratii* Garrido, Castillejo and Iglesias, 1995					
ira-559 (paratype)	Navarra, Spain	AY987892	AY947367	AJ509042	MNCN Madrid, 15.05/18705
*Arion lizarrustii* Garrido, Castillejo and Iglesias, 1995					
liz-562 (paratype)	Navarra, Spain	AY987893	AY947368	AJ509046	MNCN Madrid, 15.05/18706
ALIZ 47C*	Lizarrusti, Spain	NA	NA	AY316280	
*Arion lusitanicus* Mabille, 1868					
**lus-1613**	Feitos, Portugal	**KF305203**	**KF356224**	NA	RBINS Brussels, I.G. 32471
**lus-1631**	Currais, Portugal	**KF305204**	**KF356225**	NA	RBINS Brussels, I.G. 32471
**lus-1641**	Cacia, Portugal	**KF305205**	NA	NA	RBINS Brussels, I.G. 32471
**lus-1647**	Cacia, Portugal	**KF305206**	NA	NA	RBINS Brussels, I.G. 32471
**lus-1652**	Forjães, Portugal	**KF305207**	**KF356226**	NA	RBINS Brussels, I.G. 32471
**lus-1654**	Currais, Portugal	NA	**KF356227**	NA	RBINS Brussels, I.G. 32471
**lus-1655**	Forjães, Portugal	**KF305208**	**KF356228**	NA	RBINS Brussels, I.G. 32471
lus-79	Ursel, Belgium	AY987894	AY947369	AJ509062	RBINS Brussels, I.G. 32471
**lus-181**	Terceira, Azores, Portugal	NA	NA	**KF385453**	RBINS Brussels, I.G. 32471
lus-186	Namur, Belgium	AY987895	AY947370	AJ509061	RBINS Brussels, I.G. 32471
lus-465	Görlitz, Germany	NA	NA	AJ509063	RBINS Brussels, I.G. 32471
**lus-509**	Emptinne, Belgium	**KF305209**	**KF356229**	NA	RBINS Brussels, I.G. 32471
ALUS 42A*	Serra de Arrábida, Portugal	NA	NA	AY316273	
ALUS 42B*	Serra de Arrábida, Portugal	NA	NA	AY316274	
ALUS 42C*	Serra de Arrábida, Portugal	NA	NA	AY316275	
ALUS 42G*	Alpi Carniche, Rivolato, Italy	NA	NA	AY316276	
ALUS 62E*	Montagne Noire, France	NA	NA	AY316289	
ALUS 70C*	Girona, Spain	NA	NA	AY316290	
*Arion molinae* Garrido, Castillejo and Iglesias, 1995					
mol-565 (paratype)	La Molina, Spain	AY987896	AY947371	AJ509043	MNCN Madrid, 15.05/18707
AMOL 48A*	Serra del Cadí, Barcelona, Spain	NA	NA	AY316281	
*Arion nobrei* Pollonera, 1889					
ANOB 41A*	Luso, Portugal	NA	NA	AY316271	
ANOB 41B*	Luso, Portugal	NA	NA	AY316272	
*Arion paularensis* Wiktor and Parejo, 1989					
pau-121	Sierra de Guadarrama, Spain	**KF305210**	NA	**KF385454**	RBINS Brussels, I.G. 32471
pau-224	Sierra de Guadarrama, Spain	AY987899	AY947374	AJ509057	RBINS Brussels, I.G. 32471
pau-226	Sierra de Guadarrama, Spain	NA	NA	**KF385455**	RBINS Brussels, I.G. 32471
APAU 51A*	Sierra de Guadarrama, Spain	NA	NA	AY316284	
*Arion ponsi* Quintana Cardona, 2007					
**pon-1959**	Ciutadella de Menorca, Spain	**KF305211**	**KF356230**	**KF385456**	RBINS Brussels, I.G. 32471
**pon-1960**	Ferreries, Menorca, Spain	**KF305212**	**KF356231**	**KF385457**	RBINS Brussels, I.G. 32471
pon-1962	Ciutadella de Menorca, Spain	**KF305213**	**KF356232**	**KF385458**	RBINS Brussels, I.G. 32471
**pon-1965**	Ciutadella de Menorca, Spain	**KF305214**	**KF356233**	**KF385459**	RBINS Brussels, I.G. 32471
*Arion subfuscus* (Draparnaud, 1805)					
**sub1-2312**	Kortrijk, Belgium	**KF305215**	**KF356238**	**KF385461**	RBINS Brussels, I.G. 32471
sub1-2318	Ingrandes, France	AY987904	AY860678	AY860729	RBINS Brussels, I.G. 32471
sub1-2317	Burnopfield, UK	AY987906	AY860672	AY860726	RBINS Brussels, I.G. 32471
**sub1-SU87**	Barnstable, MA, USA	NA	KF356235	NA	RBINS Brussels, I.G. 32471
**sub1-1618**	Wilrijk, Belgium	**KF305216**	**KF356236**	NA	RBINS Brussels, I.G. 32471
**sub1-1633**	Wilrijk, Belgium	**KF305217**	**KF356237**	NA	RBINS Brussels, I.G. 32471
**sub2-SU2424**	Heppenbach, Belgium	NA	**KF356239**	NA	RBINS Brussels, I.G. 32471
**sub2-SU349**	Grootrees, Belgium	NA	**KF356240**	NA	RBINS Brussels, I.G. 32471
**sub2-2309**	Gomzé, Belgium	**KF305218**	**KF356241**	**KF385462**	RBINS Brussels, I.G. 32471
**sub2-2313**	Le Landin, France	AY987908	AY860687	**KF385463**	RBINS Brussels, I.G. 32471
**sub2-2314**	Heppenbach, Belgium	AY987909	AY860709	**KF385464**	RBINS Brussels, I.G. 32471
**sub3-2322**	Bucholz, Germany	AY987910	AY860716	**KF385466**	RBINS Brussels, I.G. 32471
**sub3-2310**	La Salle, France	AY987911	AY860722	**KF385465**	RBINS Brussels, I.G. 32471
**sub3-SU2401**	La Salle, France	NA	**KF356242**	NA	RBINS Brussels, I.G. 32471
sub4-123 (topotype)	Montagne Noire, France	AY987913	AY860682	AY860733	RBINS Brussels, I.G. 32471
sub4-568 (neotype)	Montagne Noire, France	AY987914	**KF356244**	AJ509044	MNCN Madrid, 15.05/18704
sub4-2341	Oulès, France	AY987912	AY860685	AY860731	RBINS Brussels, I.G. 32471
**sub4-SU1058**	Col de Peyresourde, France	NA	**KF356243**	NA	RBINS Brussels, I.G. 32471
**sub5-2321**	Villemont-Baubiat, France	AY987915	AY860681	**KF385468**	RBINS Brussels, I.G. 32471
**sub5-2311**	Villemont-Baubiat, France	AY987916	AY860679	**KF385467**	RBINS Brussels, I.G. 32471
ASUB 45A*	Montagne Noire, France	NA	NA	AY316279	
*Arion transsylvanus* Simroth, 1885					
tra-SU1088	Covasna, Romania	AY943858	AY860798	AY947393	RBINS Brussels, I.G. 30412
tra-SU1203	Lunca Vişagului, Romania	AY943859	AY860805	AY947394	RBINS Brussels, I.G. 30412
tra-SU1296	Holda, Romania	AY943860	AY860799	AY947395	RBINS Brussels, I.G. 30412
*Arion urbiae* De Winter, 1986					
urb-SU2755	Saldaña, Spain	AY987919	AY947381	AY947396	RBINS Brussels, I.G. 32471
**urb-99**	Sierra da Urbia, Spain	NA	NA	**KF385469**	RBINS Brussels, I.G. 32471
AURB 50A*		NA	NA	AY316283	
**Subgenus *Kobeltia* Seibert, 1873**					
*Arion distinctus* Mabille, 1868					
dis-106	Mortsel, Belgium	AY987875	AY947354	AJ509040	RBINS Brussels, I.G. 32471
dis-14		AY987874	AY947353	AJ509038	RBINS Brussels, I.G. 32471
*Arion hortensis* Férussac, 1819					
hor-102	Mortsel, Belgium	AY987888	AJ518061	AJ509037	RBINS Brussels, I.G. 32471
hor-220	London, UK	AY987889	AY947364	AJ509036	RBINS Brussels, I.G. 32471
*Arion intermedius* Normand, 1852					
int-104	Rochefort, Belgium	AY987891	AY947366	AJ509031	RBINS Brussels, I.G. 32471
int-52	Flores, Azores, Portugal	AY987890	AY947365	AJ509029	RBINS Brussels, I.G. 32471
*Arion obesoductus* Reischütz, 1973					
alp-1610	Žďárské Vrchy, Czech Republic	DQ904249	DQ904248	NA	RBINS Brussels, I.G. 32471
alp-208	Saxony, Germany	AY987867	AY947346	AJ509041	RBINS Brussels, I.G. 32471
*Arion owenii* Davies, 1979					
owe-310	Devon, UK	AY987897	AY947372	AJ509033	RBINS Brussels, I.G. 32471
owe-316	Devon, UK	AY987898	AY947373	AJ509034	RBINS Brussels, I.G. 32471
*Arion wiktori* Parejo & Martín, 1990					
wik-SU2693	Viniegra de Abajo, Spain	AY987921	AY947383	AY947397	RBINS Brussels, I.G. 32471
wik-44	Burgos, Spain	AY987920	AY947382	AJ509060	RBINS Brussels, I.G. 32471
**wik-94**	Burgos, Spain	NA	**KF356245**	AJ509059	RBINS Brussels, I.G. 32471
AWIK 58A*	Demanda Sierra, Burgos, Spain	NA	NA	AY316287	
AWIK 58C*	Urbión Mountains, Soria, Spain	NA	NA	AY316288	
**Subgenus *Carinarion* Hesse, 1926**					
*Arion circumscriptus* Johnston, 1828					
cir-151	Aran Island, Kilmurvey, Ireland	AY987872	AY947351	AJ509071	RBINS Brussels, I.G. 32471
*Arion fasciatus* (Nilsson, 1823)					
fas-144	Görlitz, Germany	AY987877	AY947356	AJ509068	RBINS Brussels, I.G. 32471
*Arion silvaticus* Lohmander, 1937					
sil-142	Poulseur, Belgium	AY987917	AY947379	AJ509070	RBINS Brussels, I.G. 32471
**Subgenus *Arion* s.s. Férussac, 1819**					
*Arion ater-rufus* complex					
ate-SU517	Musland, Norway	AY987870	AY947349	AY947387	RBINS Brussels, I.G. 32471
**ate/ruf-1602**	Manteigas, Portugal	**KF305219**	NA	**KF385449**	RBINS Brussels, I.G. 32471
**ate/ruf-1619**	Santa Leocádia, Portugal	**KF305220**	**KF356213**	NA	RBINS Brussels, I.G. 32471
**ate/ruf-1620**	Gortmore, Ireland	**KF305221**	**KF356214**	NA	RBINS Brussels, I.G. 32471
**ate/ruf-1624**	Oleirinhos, Portugal	**KF305222**	**KF356215**	NA	RBINS Brussels, I.G. 32471
**ate/ruf-1638**	Portulezo, Portugal	**KF305223**	**KF356216**	NA	RBINS Brussels, I.G. 32471
**ate/ruf-1649**	Manteigas, Portugal	**KF305224**	**KF356217**	NA	RBINS Brussels, I.G. 32471
**ruf-105**	St.-Katelijne Waver, Belgium	**KF305225**	**KF356234**	NA	RBINS Brussels, I.G. 32471
ruf-15	Santiago de Compostela, Spain	AY987900	AY947375	AJ509066	RBINS Brussels, I.G. 32471
ruf-155	Brussels, Belgium	AY987901	AY947376	AJ509064	RBINS Brussels, I.G. 32471
ruf-180	Hoboken, Belgium	AY987902	AY947377	AJ509065	RBINS Brussels, I.G. 32471
ruf-182	Brecht, Belgium	AY987903	AY947378	AJ509067	RBINS Brussels, I.G. 32471
**ruf-624**	Nazareth, Belgium	NA	NA	KF385460	RBINS Brussels, I.G. 32471
AATE 39A*	Caldas de Gerês, Portugal	NA	NA	AY316268	
AATE 39E*	Valporquero Cave, Leon, Spain	NA	NA	AY316269	
ARUF 40G*	Montagne Noire, France	NA	NA	AY316270	

Sequences were aligned in ClustalW (Thompson et al. 1994) with default settings and without subsequent manual adjustments. In each alignment sequences were trimmed to equal length. The final alignments had a length of 504 bp (COI), 408 bp (16S) and 587 bp (ITS1), and of 1499 bp after concatenating the three fragments. The COI sequences were translated to amino acid sequences to check for stop codons (but none were found). The ITS1 sequences were also analysed together with those of Quinteiro et al. (2005). In this way we could extend our taxon coverage to *Arion hispanicus* Simroth, 1886, *Arion fuligineus* Morelet, 1845 and *Arion nobrei* Pollonera, 1889 (Table 1). Because Quinteiro et al. (2005) used other ITS1 primers, we had to trim this dataset to a length of 378 bp. For each gene fragment, and for the concatenated dataset, we constructed Neighbour-Joining (NJ) trees (Saitou and Nei 1987) using the Kimura 2-parameter (K2P) model in MEGA v5 (Tamura et al. 2011) with complete deletion of insertions and deletions (indels). Branch support was evaluated with 1000 bootstrap replicates (Felsenstein 1985). Only bootstrap values ≥ 70% were considered as indicating strong support (Hillis and Bull 1993). Uncorrected p-distances (hereafter simply referred to as p-distance) were calculated in MEGA v5 (Tamura et al. 2011). For these calculations we considered the following Molecular Operational Taxonomic Units (MOTUs): 1) the five 16S rDNA clades of *Arion subfuscus* (S1 to S5) defined by Pinceel et al. (2005a), 2) *Arion anguloi* and *Arion urbiae* jointly as a single MOTU (Backeljau et al. 1994, Quinteiro et al. 2005), 3) *Arion wiktori* and *Arion paularensis* jointly as a single MOTU (Backeljau et al. 1996, Quinteiro et al. (2005), and 4) *Arion lusitanicus* from Portugal vs. *Arion lusitanicus* from elsewhere as two different MOTUs (Davies 1987, Castillejo 1998, Quinteiro et al. 2005). Standard errors of mean p-distances among taxa and MOTUs were calculated on 1000 bootstrap replicates.

## Results

### Overall

**Figure 3.**
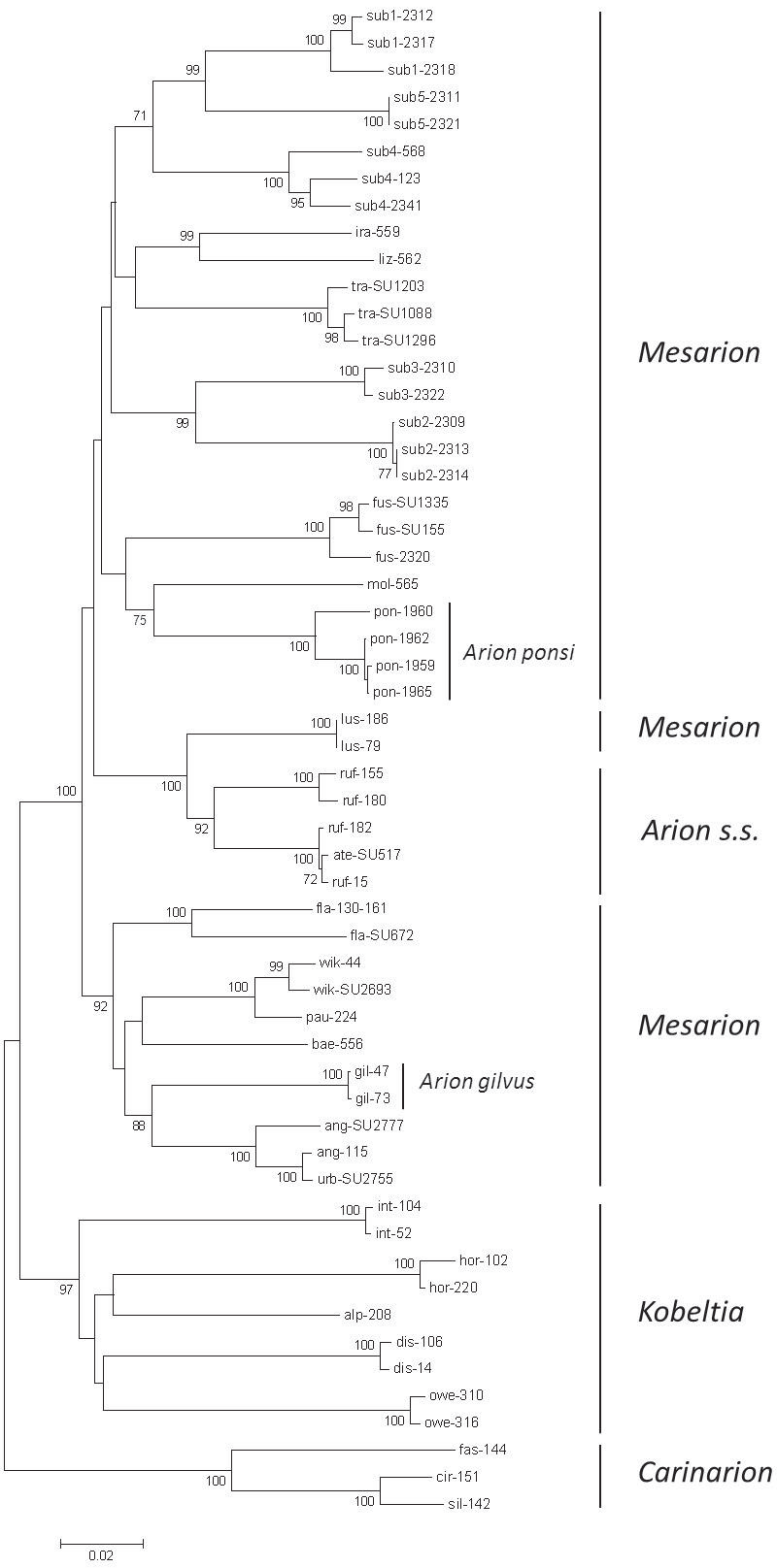
Neighbour-Joining tree (Kimura 2-parameter model) of a 1499 bp concatenated fragment (504 bp of the mitochondrial cytochrome *c* oxidase subunit I (COI) gene, 408 bp of the mitochondrial 16S rDNA gene, 587 bp fragment of the nuclear internal transcribed spacer 1 (ITS1) region) for the land slug subgenus *Mesarion*. Bootstrap values ≥ 70% are shown at the nodes. For sample codes see Table 1.

**Supplementary figure 1.**
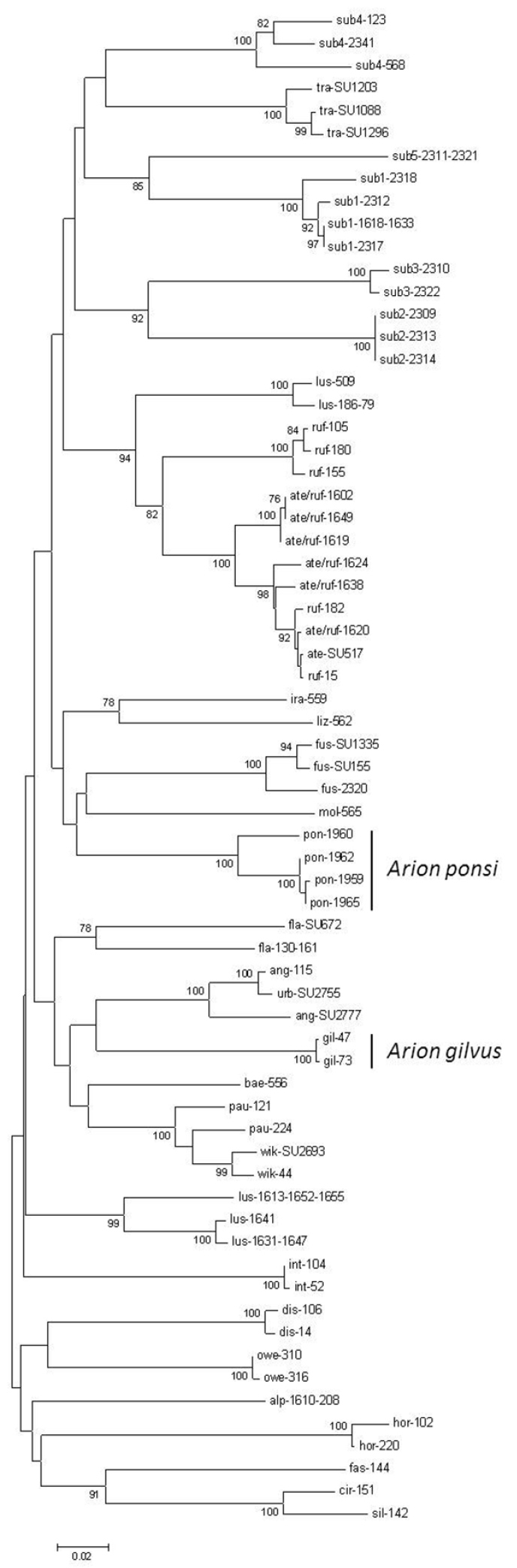
Neighbour-Joining tree (Kimura 2-parameter model) of a 504 bp fragment of the mitochondrial cytochrome *c* oxidase subunit I (COI) gene for the land slug subgenus *Mesarion*. Bootstrap values ≥ 70% are shown at the nodes. For sample codes see Table 1.

**Supplementary figure 2.**
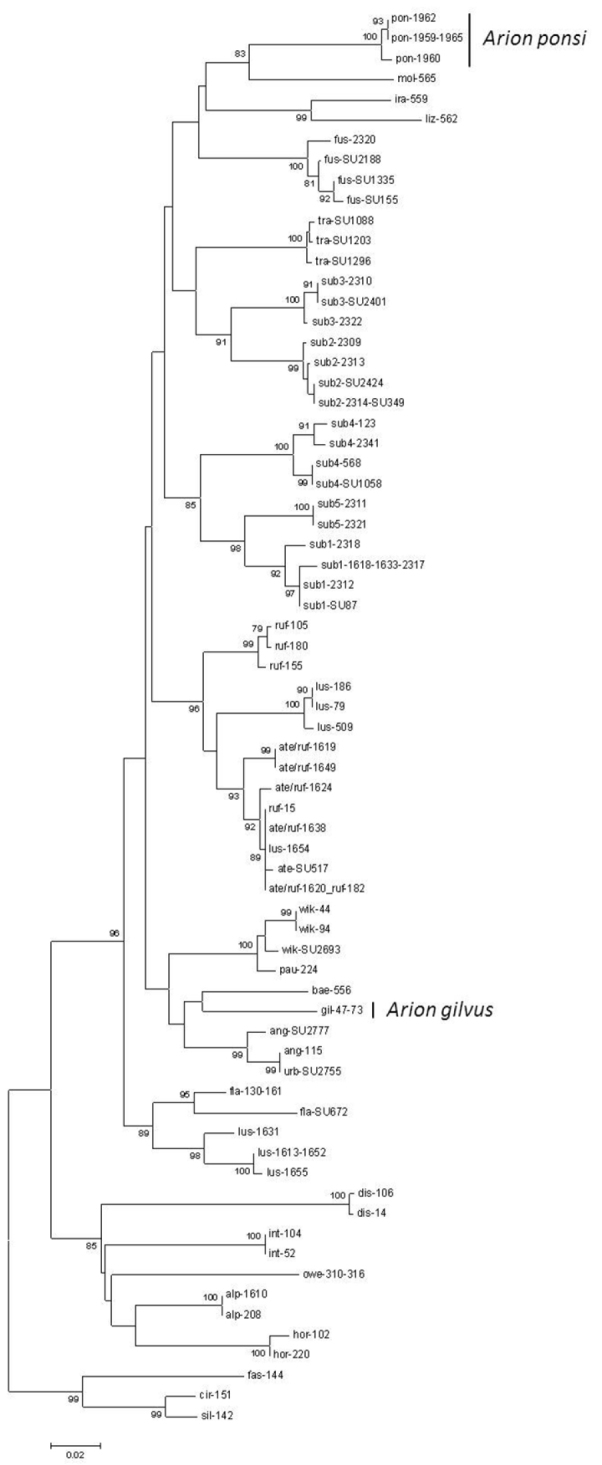
Neighbour-Joining tree (Kimura 2-parameter model) of a 408 bp fragment of the mitochondrial 16S rDNA gene for the land slug subgenus *Mesarion*. Bootstrap values ≥ 70% are shown at the nodes. For sample codes see Table 1.

**Supplementary figure 3.**
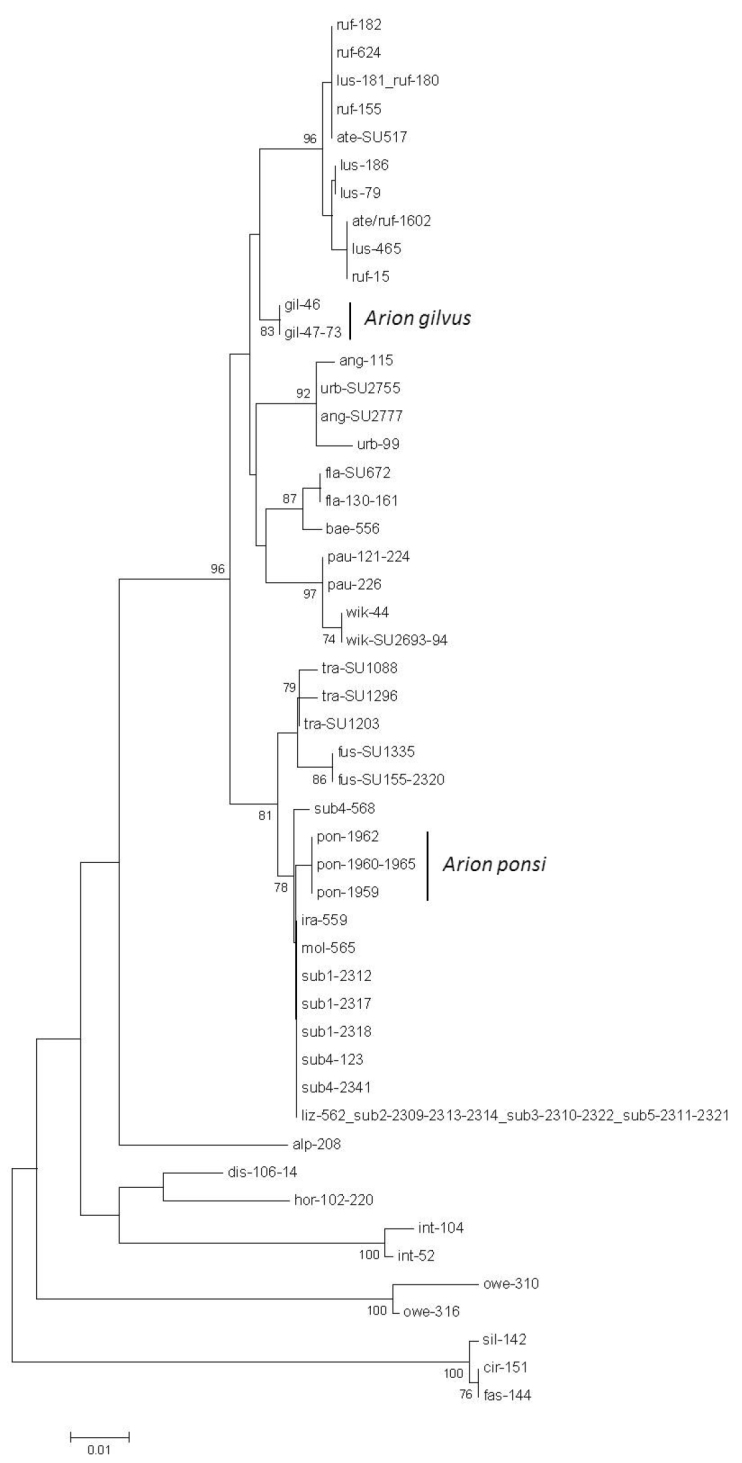
Neighbour-Joining tree (Kimura 2-parameter model) of a 587 bp fragment of the nuclear internal transcribed spacer 1 (ITS1) region for the land slug subgenus *Mesarion*. Bootstrap values ≥ 70% are shown at the nodes. For sample codes see Table 1.

**Supplementary figure 4.**
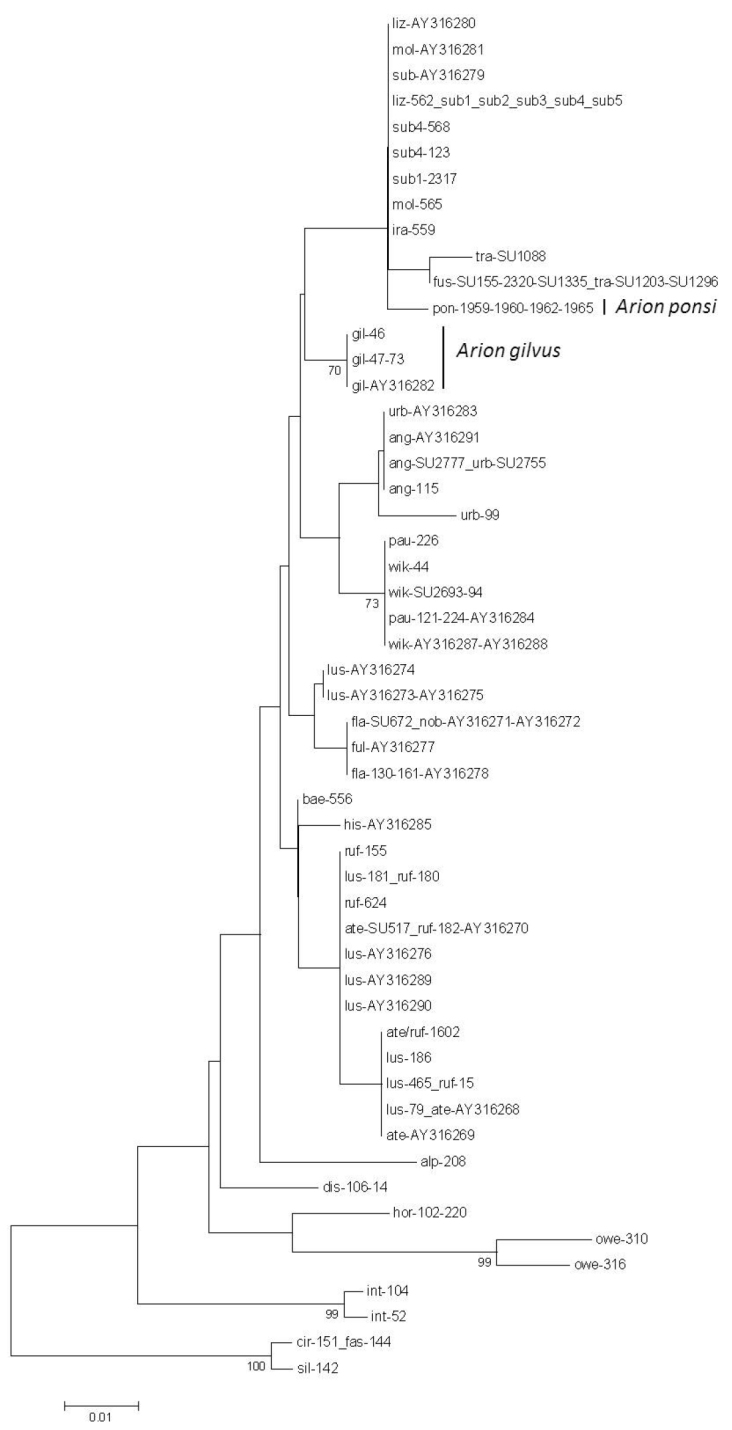
Neighbour-Joining tree (Kimura 2-parameter model) of a 378 bp fragment of the nuclear internal transcribed spacer 1 (ITS1) region for the land slug subgenus *Mesarion*. This figure also includes the Iberian *Mesarion* ITS1 sequences of Quinteiro et al. (2005) Bootstrap values ≥ 70% are shown at the nodes. For sample codes see Table 1.

The alignments comprized 504 bp for COI (196 variable sites), 408 bp for 16S (121 sites with alignment gaps, 122 variable sites) and 587 bp for ITS1 (277 sites with alignment gaps, 64 variable sites). For the concatenated dataset, there was strong support for the subgenera *Carinarion*, *Kobeltia* (excluding *Arion wiktori*) and *Arion* s.s., and for a clade of *Arion* s.s. + *Mesarion* (including *Arion wiktori*) (Figure 3). The subgenus *Mesarion* was not monophyletic but consisted of (1) a clade of *Arion flagellus*, *Arion wiktori*, *Arion paularensis*, *Arion baeticus*, *Arion urbiae*, *Arion anguloi*, and *Arion gilvus*, (2) two haplotypes of *Arion lusitanicus* (lus-79 and lus-186) that formed a sister group of *Arion* s.s. [insofar *Arion lusitanicus* is, of course, considered as a member of *Mesarion*; see e.g. Backeljau (1989)], and (3) a number of species/clades among which the relationships were mostly unresolved. Within *Arion subfuscus* (for which the monophyly was not supported) there were five clades (S1 to S5), with strong support for (S1,S5),S4) and (S2,S3). The mean p-distance (± SE) among the *Mesarion* OTUs (including *Arion ponsi* and *Arion gilvus*) was 0.168 ± 0.011 (range: 0.11–0.22) for COI, 0.134 ± 0.012 (range: 0.058–0.195) for 16S, and 0.022 ± 0.004 (range: 0.000–0.048) for ITS1 (a minimum distance of zero means that the two sequences only differed in a number of indels). The mean p-distances (± SE) excluding *Arion ponsi* and *Arion gilvus* were 0.167 ± 0.011 (range: 0.11–0.22) for COI, 0.130 ± 0.012 (range: 0.058–0.195) for 16S, and 0.023 ± 0.004 (range: 0.000–0.048) for ITS1. For the concatenated dataset these values were 0.108 ± 0.006 (range: 0.071–0.137) (including *Arion ponsi* and *Arion gilvus*) and 0.107 ± 0.006 (range: 0.071–0.137) (excluding *Arion ponsi* and *Arion gilvus*). The phylogenetic trees inferred from the three gene fragments and from the concatenated dataset are shown in Appendix, Supplementary figures 1–4 and Figure 3, respectively.

### Arion ponsi

The four individuals of *Arion ponsi* yielded four COI and three 16S haplotypes (Appendix, Supplementary figures 1–2), yet two 16S haplotypes only differed by an indel of two base pairs at positions 291–292. For both genes *Arion molinae* showed the smallest p-distance with *Arion ponsi* (COI: mean p-distance 0.142 ± 0.014; 16S: mean p-distance 0.162 ± 0.019), but a sister species relationship with *Arion molinae* was only well-supported by 16S. There were three ITS1 haplotypes for *Arion ponsi*; one of these had a deletion of a poly-T stretch of six base pairs at positions 556–561; the other two differed by a deletion of a G at position 554. These three ITS1 haplotypes of *Arion ponsi* clustered within a clade of *Arion subfuscus* S1–5, *Arion lizarrustii*, *Arion molinae*, *Arion iratii* and *Arion transsylvanus* (Appendix, Supplementary figure 3). The ITS1 analysis with the sequences of Quinteiro et al. (2005), placed the single remaining *Arion ponsi* haplotype in the same clade (mean p-distance with the other taxa of this clade = 0.046 ± 0.004), but without bootstrap support (Appendix, Supplementary figure 4).

As for 16S, the concatenated tree of the three gene fragments showed a sister species relationship between *Arion ponsi* and *Arion molinae* (Figure 3).

### Arion gilvus

The three *Arion gilvus* specimens yielded two COI (one synonymous A-G substitution at position 366) and one 16S haplotypes. For both genes the smallest mean p-distances were observed relative to *Arion urbiae* and *Arion anguloi* (COI: mean p-distance = 0.145 ± 0.013; 16S: mean p-distance = 0.134 ± 0.016). The two *Arion gilvus* ITS1 haplotypes reduced to one when considering the stretch that overlapped with the Quinteiro et al. (2005) sequences. In this stretch it differed from that of Quinteiro et al. (2005) by a deletion of a T at position 349. Separately, none of the three genes provided reliable evidence about the sister group relationships of *Arion gilvus* (Appendix, Supplementary figures 1–4). Yet, the concatenated tree showed a well-supported sister species relationship between *Arion gilvus* and the *Arion urbiae* / *Arion anguloi* clade (mean p-distance = 0.021 ± 0.003) (Figure 3). Mean p-distances within this *Arion urbiae* / *Arion anguloi* clade (in which *Arion anguloi* was paraphyletic) were p = 0.041 ± 0.006 for COI, p = 0.023 ± 0.006 for 16S, p = 0.004 ± 0.002 for ITS1 and p = 0.020 ± 0.003 for the concatenated dataset.

## Discussion

The NJ-tree of the concatenated dataset confirms the major outcomes of previous phylogenetic studies, viz. 1) a strong support for the monophyly of the subgenus *Carinarion* (Geenen et al. 2006), 2) a clade of *Arion* s.s. and non-Portuguese *Arion lusitanicus* (Quinteiro et al. 2005), 3) *Arion wiktori* clustering with *Mesarion* species, in particular with *Arion paularensis* (Quinteiro et al. 2005) instead of with *Kobeltia* species (Castillejo 1998), and 4) the strong differentiation within *Arion subfuscus* s.s. that consists of, at least, five phylogenetic species (Pinceel et al. 2005a). It therefore seems that the analysis of COI, 16S and ITS1 DNA sequences yields relevant taxonomic information with respect to the characterisation of arionid species that have been described under the morphospecies concept.

Because *Arion gilvus* and *Arion ponsi* were originally described on morphological grounds they are to be interpreted as morphospecies. This phenetic morphological distinction, however, correlates well with a phenetic separation based on mtDNA distances. Indeed, the overall mean p-distance among the *Mesarion* MOTUs (excluding *Arion ponsi* and *Arion gilvus*) dealt with in this study is 16.7% for COI and 13% for 16S. As such, the mean p-distances between *Arion ponsi* and *Arion molinae* (COI: 14.2%; 16S: 16.2%) or between *Arion gilvus* and *Arion urbiae* (COI: 14.5%; 16S: 13.4%) are perfectly comparable with the mean p-distances among the other MOTUs and morphospecies in *Mesarion*. Hence, with respect to mtDNA divergence, both *Arion ponsi* and *Arion gilvus*, behave as other *Mesarion* species or putative species-level MOTUs.

Obviously, the strong COI differentiation among *Mesarion* taxa, and of *Arion ponsi* and *Arion gilvus* in particular, suggests that DNA barcoding may be a suitable identification tool for these animals. Yet, this may be a too simplistic conclusion, since stylommatophorans may show extremely high intraspecific mtDNA divergences of sometimes up to 27% (K2P-distances, but note the uncorrected p-distances are almost similar) (Thomaz et al. 1996, Chiba 1999). In addition, Davison et al. (2009) showed that in the Stylommatophora the mean interspecific K2P-distances (± 3%) can be substantially lower than the mean intraspecific K2P-distances (± 12%). Under these conditions, it becomes very difficult to define generally applicable thresholds that distinguish between intra- and interspecific sequence divergences. Such thresholds are normally associated with DNA barcoding gaps (Hebert et al. 2003), but Davison et al. (2009) were unable to detect DNA barcoding gaps in the taxa they studied. Nevertheless, Davison et al. (2009) suggested a pragmatic 4% threshold to separate intra- and interspecific values, but at the same time they also concluded that DNA barcoding in itself is insufficient to identify and/or detect stylommatophoran species. Unfortunately, our sample sizes were too small to explore eventual DNA barcoding gaps in *Mesarion*.

Because DNA barcoding on its own may be unreliable for identifying and detecting species-level taxa in stylommatophorans, it its necessary to backup this sort of data with, amongst others, phylogenetic analyses. As such, our phylogenetic trees of the DNA sequence data show that the morphospecies *Arion ponsi* and *Arion gilvus*, also represent phylogenetic species, since both form well-supported clades that are “significantly” associated with well-defined, but morphologically different sister species. For *Arion ponsi*, the sister species appears to be *Arion molinae*, the distribution range of which is located in NE continental Spain (Castillejo 1997), i.e. north of, and facing, the Balearic Islands. Conversely, the sister taxon of *Arion gilvus* is the “tandem” of *Arion urbiae* and *Arion anguloi*, two species that have been synonymized by Backeljau et al. (1994) and that jointly should be referred to as *Arion urbiae*. Our DNA sequence data on COI, 16S and ITS1 (e.g. Figure 3), as well as those on ND1 and ITS1 of Quinteiro et al. (2005) are in line with this. As such, the distribution range of *Arion urbiae* is situated northwest of, and probably adjacent to, that of *Arion gilvus*. Thus, for both the species pairs *Arion ponsi* / *Arion molinae* and *Arion gilvus* / *Arion urbiae*, the distribution ranges appear at least consistent with the suggested sister group relationships.

In conclusion, the present work shows that *Arion ponsi* and *Arion gilvus* clearly differ from *Arion subfuscus* or any other currently recognized arionid species. As such, former records of *Arion subfuscus* from Menorca (e.g. Gasull and van Regteren Altena 1970, Mateo 1993, Beckmann 2007) almost certainly refer to *Arion ponsi*. Similarly, probably all reports of *Arion subfuscus* in the regions of Valencia and Albacete involve *Arion gilvus* (e.g. Borredà 1994, Borredà and Collado 1996). Finally, Borredà (1994) wondered about the eventual relationship between *Arion subfuscus* from Menorca and *Arion gilvus*. The current data confirm unambiguously that these are two different species, with the former being *Arion ponsi*. Yet, the overall phylogenetic relationships within *Mesarion* and many other *Arion subfuscus*-like taxa remain to be resolved. In this context, one of the main questions is whether *Mesarion* in its present use is a monophyletic taxon. At the same time one may wonder about the relationships with the subgenus *Arion* s.s., with which *Mesarion* seems to form a well-supported clade (Figure 3).
